# Transcriptional Profile of Muscle following Acute Induction of Symptoms in a Mouse Model of Kennedy's Disease/Spinobulbar Muscular Atrophy

**DOI:** 10.1371/journal.pone.0118120

**Published:** 2015-02-26

**Authors:** Katherine Halievski, Kaiguo Mo, J. Timothy Westwood, Douglas A. Monks

**Affiliations:** Department of Psychology, University of Toronto Mississauga, Mississauga, Ontario, Canada; University of Edinburgh, UNITED KINGDOM

## Abstract

**Background:**

Kennedy’s disease/Spinobulbar muscular atrophy (KD/SBMA) is a degenerative neuromuscular disease affecting males. This disease is caused by polyglutamine expansion mutations of the androgen receptor (*AR*) gene. Although KD/SBMA has been traditionally considered a motor neuron disease, emerging evidence points to a central etiological role of muscle. We previously reported a microarray study of genes differentially expressed in muscle of three genetically unique mouse models of KD/SBMA but were unable to detect those which are androgen-dependent or are associated with onset of symptoms.

**Methodology/Principal Findings:**

In the current study we examined the time course and androgen-dependence of transcriptional changes in the HSA-AR transgenic (Tg) mouse model, in which females have a severe phenotype after acute testosterone treatment. Using microarray analysis we identified differentially expressed genes at the onset and peak of muscle weakness in testosterone-treated Tg females. We found both transient and persistent groups of differentially expressed genes and analysis of gene function indicated functional groups such as mitochondrion, ion and nucleotide binding, muscle development, and sarcomere maintenance.

**Conclusions/Significance:**

By comparing the current results with those from the three previously reported models we were able to identify KD/SBMA candidate genes that are androgen dependent, and occur early in the disease process, properties which are promising for targeted therapeutics.

## Introduction

Kennedy's Disease/Spinobulbar muscular atrophy (KD/SBMA) is an X-linked, androgen-dependent neuromuscular disease caused by polyglutamine (polyQ) mutation of the androgen receptor (*AR*) gene [1,2]. KD/SBMA is characterized by a late-onset, progressive weakness of proximal limbs and bulbar muscles in affected men as well as androgen insensitivity (such as gynecomastia and infertility) and some sensory deficits. Though this is predominately considered a “motor neuron disease”, myogenic origins have been proposed (reviewed in [3,4–7]). Evidence of myopathy prior to motor neuron pathology (e.g., [8]) indicates that AR may first cause dysfunction in muscles, which then leads to dysfunction of the motor neurons that innervate them. Similarly, selective AR overexpression limited to muscle is sufficient to cause motor neuron pathology [9]. Moreover, a recent study in a KD/SBMA mouse model demonstrated that removing mutant AR solely from muscles is sufficient to improve motor function and survival [10]. Since the motor unit (motor neuron and muscle fibre) is interdependent, it is of interest to determine which pathological actions of *AR* can occur in muscles as this may give insight into the overall disease process in KD/SBMA.

We previously reported a microarray study of gene expression in muscle of three different mouse models of KD/SBMA [11]. Briefly, the three male KD/SBMA mouse models we used for comparison were: a polyQ expanded AR knock-in model (AR113Q KI; [8]); a transgenic (Tg) model that overexpresses polyQ AR ubiquitously (AR97Q Tg; [12]); and a Tg model that overexpresses wild-type (WT) AR solely in muscle (HSA-AR Tg; [9]). Identifying transcriptional changes in muscle in mouse models of KD/SBMA helps to identify candidate therapeutic targets. Importantly, a common pattern of altered gene expression was observed in these independently generated models, each with a distinct genetic basis. This common pattern of gene expression raises the possibility of identifying genes which are sufficient to cause KD/SBMA symptoms. Elucidating the molecular bases of KD/SBMA is of great potential benefit as there is currently no treatment for the disease. Although the previous study greatly narrowed down the list of candidate genes, we were unable resolve which genes are androgen-dependent nor were we able to distinguish between genes that are associated with the onset of the disease and those that are associated with later stages of the disease process and may therefore be involved with compensation or degeneration.

In this paper we present a study of transcriptional changes that occur early and late in motor dysfunction progression and, importantly, are androgen-dependent. To do so, we elected to use one of the models of KD/SBMA used in our previous study, which has the unique advantage of having a severe phenotype following acute androgen treatment in females. This HSA-AR Tg mouse model overexpresses wild-type AR exclusively in myocytes [9]. The HSA-AR model strongly reproduces the sex limited (i.e., male) and androgen dependent features of the KD/SBMA phenotype. Treating non-symptomatic females with testosterone (T) induces disease symptoms within 3 days (d) and severe symptoms typical of diseased males are seen by 7d. Using microarray analysis of gene expression within muscle from both Tg and WT females T-treated for 3 or 7d we are therefore able to determine which candidate genes are androgen dependent, and which are associated with the onset and later progression of KD/SBMA in this mouse model.

## Materials and Methods

### Animals

Ten WT C57BL/6J mice (5 males and 5 females, 70d old, Jackson Laboratories) were used to make the RNA reference samples. Fifteen female HSA-AR mice from Line 141 (6 Tg, 9 WT; 120–200d old) and 5 male HSA-AR Tg line 141 mice (110–130d of age) were used in this study. The production, genotyping, and phenotyping of HSA-AR transgenic mice has been described previously [9]. All animal experiments conformed to NIH guidelines and were approved by the University Animal Care Committee of the University of Toronto Mississauga (Approved protocol #20007262).

Five sample groups of 3 animals each were performed: Tg female 3d T treatment; Tg female 7d T treatment; WT untreated; WT 3d T treatment; and WT 7d T treatment. We additionally used microarray data from HSA-AR males [11] to compare with HSA-AR females.

### Testosterone Treatment and Animal Surgery

Briefly, all female mice (Tg or WT) used in this study were ovariectomized under isoflurane anesthesia and received subcutaneous Silastic implants that were either empty or filled with crystalline T (1.57mm inner diameter and 3.18mm outer diameter; effective release length of 6mm; for more detail see [13]). Such T implants result in low physiological levels of T similar to those found in adult males [13]. After 3 or 7d of T treatment, mice were put under surgical anesthesia and all hindlimb muscles (where most mass comes from quadriceps) were harvested and immediately frozen in liquid nitrogen before storage at -80°C.

### Total RNA Preparation

Frozen limb muscles were placed in TRizol Reagent (Invitrogen Corporation, Carlsbad, CA) and homogenized before RNA extraction. The total RNA extraction was performed according to the manufacturer’s guidelines. After purification, the RNA concentrations were determined; the *A260/A280* and *A260/A230* ratios were calculated as indices of protein and volatile compound contamination, respectively, using a spectrophotometer (NanoDrop ND-1000; ThermoScientific). The integrity of the total RNA was determined by electrophoresis of glyoxylated RNA through 1.2% agarose gel and visualization by staining with ethidium bromide. Total RNA was then used for microarray analysis and quantitative RT-PCR experiments.

### Sample Labeling and Microarray Hybridization

Two-color microarray experiments were performed using 38.5K oligo mouse MEEBO arrays (Mouse Ready Arrays, Microarrays Inc., Nashville, TN). This array contains 35,302 oligonucleotide (70mer) probes, largely derived from constitutively expressed exons and represents approximately 25,000 mouse genes. Cyanine dyes were directly incorporated into cDNA synthesized from total RNA following the procedure of Neal et al. [14]. Briefly, 38l reactions containing 20g of total RNA, 500 mol/L of dATP, dGTP and dTTP; 50 mol/L dCTP (GE Life Sciences), 25 mol/L Cy3- or Cy5-dCTP (Perkin Elmer), 10 mol/L DTT and 150 pmol oligo dT20 primer were heated to 65°C for 5 min, then 42°C for 5 min. 2l SuperScript II reverse transcriptase (Invitrogen Corporation) was added, and cDNA synthesis was carried out for 3h at 42°C. Reactions were stopped by the addition of 5L of 50 mol/L EDTA. RNA was hydrolyzed with 4L of 5 mol/L NaOH for 10 min at 65°C, and the reaction was then neutralized by titration with acetic acid. The cDNA from one Cyanine-3 (Perkin Elmer, Boston, MA) reaction (reference sample) were combined with those from a Cyanine-5 (Perkin Elmer, Boston, MA) reaction (experiment sample) and were co-hybridized to oligo array. Images of the hybridized arrays were acquired using a ScanArray 4000 XL laser scanner (Perkin Elmer, Boston, MA) and fluorescent intensities from spots were quantified using GenePix 5 software (Axon Instruments, Inc., CA).

### Microarray Data Analysis

Microarray images and quantification data were then imported into GeneTraffic DUO (Stratagene, La Jolla, CA) for analysis. The data were normalized using the Lowess algorithm at the subgrid level while ignoring flagged values. After normalization of the data, lists of differentially expressed genes were obtained using GeneTraffic. A universal RNA reference sample made from WT (C57BL/6J) mice was utilized on each array. Triplicate arrays using RNA samples from the different experimental animals (i.e., 3 of Tg female with 3d T treatment, 3 of Tg female with 7d T treatment, 3 of untreated WT females, 3 of WT female with 3d T treatment, 3 of WT female with 7d T treatment) were performed. Log2 ratios of experimental samples (Cy5) versus reference RNA (Cy3) were obtained. The log2 ratios of Tg mice samples were then subtracted from log2 ratios of WT to find differentially expressed genes (3d T-treated Tg subtracted from 3d T-treated WT, 7d T-treated Tg subtracted from 7d T-treated WT). log2 ratios of T-treated WT were subtracted from log2 ratio of untreated WT. Gene lists were filtered in GeneTraffic to include only those genes that displayed at least 2-fold difference and whose coefficient of variance was less than 100% and had a p-value less than 0.05 using a T-test. Dye swap experiments were not performed as previous experiments in our lab had demonstrated that they do not appreciably alter the lists of differentially expressed genes [14].

Hierarchical cluster analysis was performed in GeneTraffic DUO using the Pearson algorithm and average linkage [15]. Cluster figures were made using the MultiExperiment Viewer (MeV) in the TM4 suite of software tools (http://www.tm4.org). All microarray data is MIAME compliant and has been deposited in GEO (accession number: GSE61886).

### Real Time Quantitative RT-PCR

A two-step approach was taken in which the initial reverse transcription was followed by the quantitative PCR amplification. After DNase I (Invitrogen Corporation, CA) treatment, DNA-free total RNA was reverse transcribed using a dT_20_VN primer (Sigma, Oakville, ON) with SuperScript II. Each RNA reaction had a control reaction without reverse transcriptase to evaluate any genomic DNA contamination. Two μl of the diluted reaction was used as template for each 25 μL RT-PCR amplification. Reactions were assembled using SYBR Green JumpStart Taq ReadyMix (Sigma, Oakville, ON) and assayed on an Mx4000 Multiplex Quantitative PCR System (Stratagene, La Jolla, CA) according to the instructions of the manufacturer. Samples were incubated at 95°C for 10 min prior to thermal cycling (40 cycles of: 95°C for 30s, 57°C for 30s, and 72°C for 30s). In order to confirm the amplification specificity and identity of the PCR products, a melting curve analysis between 55°C and 95°C was also carried out automatically with the software attached to Mx4000. The completed reactions were heated to 95°C for 1 min and cooled to 55°C and reactions were re-heated in 1°C increments back to 95°C in order to plot a dissociation curve. After exporting the ROX-normalized fluorescence measurements to Microsoft Excel, the program LinRegPCR [16], was used to determine the efficiency of each reaction. These efficiencies were used in the final calculation of fold induction from the C_t_ values and the expression of each test gene was normalized to the level of glyceraldehyse 3’ dehydrogenase (GAPDH) within each sample prior to comparisons between samples.

### Primer Design

The cDNA sequences for the genes *Gapdh, Tpm3* (Tropomyosin 3), *Ky* (Kyphoscoliosis peptidase), *Tnni1* (Troponin I), *Mss51* (MSS51 mitochondrial translational activator), *Ptgds* (Prostaglandin D2 synthase), *Lcn2* (Lipocalin 2), *Ighg2a* (immunoglobulin heavy constant gamma 2A) and *Gdnf* (Glial cell line derived neurotrophic factor) were obtained from GenBank. PCR primers were designed from the corresponding cDNA sequences using the Whitehead Institute’s Primer3 software. All oligonucleotide sequences and primer pairs were checked with OligoAnalyzer 3.0 (http://scitools.idtdna.com/Analyzer/) for secondary structure and dimer formation. Each primer and amplicon sequence was tested using the nucleotide-nucleotide BLAST alignment tool to ensure minimal similarity with any other sequence. Synthesis of all primers was performed by Invitrogen. Primer sequences used in this study can be found in S1 Table.

### Functional Classification of Differentially Expressed Genes

Functional analysis of differentially expressed genes was performed using Functional Annotation Tool. The gene bank ID of differentially expressed genes were uploaded into the Database for Annotation, Visualization and Integrated Discovery (DAVID) (http://david.abcc.ncifcrf.gov) to obtain DAVID functional annotation. The genes were classified into functional groups using GO TERM biological processes and molecular functions at levels 3 and up.

## Results

Using microarray analysis, we characterized gene expression in hindlimb muscle of HSA-AR Tg female mice following 3 and 7d T treatment relative to WT females that received either 3 or 7d T treatment, respectively. WT females T-treated for 3 and 7d were compared to untreated WT females. Genes that displayed at least 2-fold difference from their comparison WT female group and *p*-value smaller than 0.05 were identified. Complete gene lists and fold changes are presented in S2 Table for Tg and S3 Table for WT mice).

We then performed hierarchical cluster analysis using these results in order to evaluate both T dependence of alterations in gene expression in skeletal muscle of KD/SBMA models and also to evaluate changes in gene expression that occur early in the disease progression. Tg females begin losing motor ability 3d following T treatment and at 7d are comparable to Tg males [9,13]. In contrast, no motor deficits or histopathology results from this treatment in WT females. We can therefore compare the time course of T responsive genes in WT and Tg females to identify candidate genes involved in producing motor deficits characteristic of KD/SBMA as well as rule out genes which are T differentially expressed in healthy female mice.

**Fig 1:**
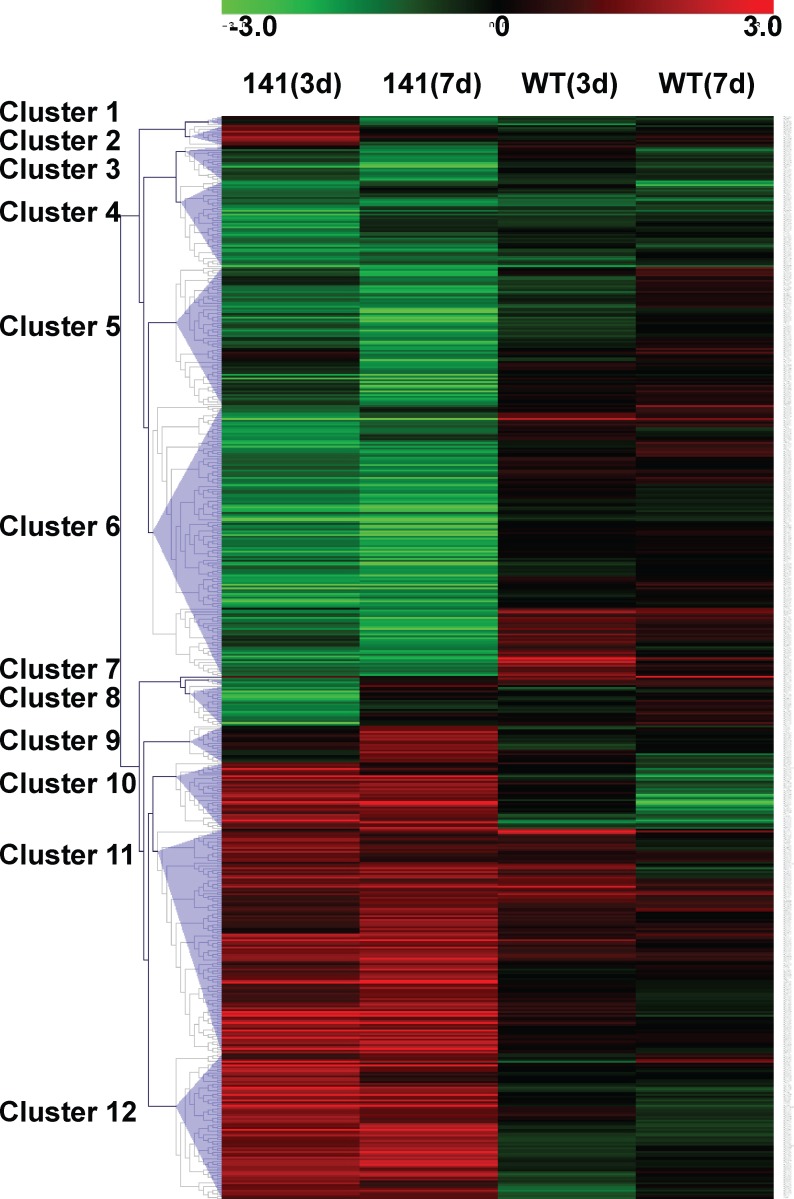
Hierarchical cluster analysis of gene expression in female HSA-AR Tg and WT mice after 3 and 7 days testosterone treatment. Cluster output represents colorimetrically indicated log2 ratio change. Female HSA-AR Tg and WT mice after 3 and 7 days testosterone treatment are represented by columns and rows represent a single gene. Clusters of genes differentially expressed in a similar pattern are labeled.

The hierarchical cluster analysis of genes that displayed at least a 2-fold difference from similarly T-treated WT females and had a *p*<0.05 (Fig. 1) generated 12 distinguishable clusters. As compared to WT mice with the same T treatment, downregulated genes were found in Tg mice after 3 and 7d of T treatment in clusters 5, 6 and upregulated genes were found in clusters 9, 10, 12 (Fig. 1). Generally, T-treated WT females did not show differential expression of most genes, indicating that the disease allele in addition to T treatment, and not T treatment alone, is playing a major role in altering transcription. Clusters 2 (upregulated) and 8 (downregulated) contain genes that are differentially expressed in 3d, but not 7d treated females, suggesting that those genes may be important in *initiating* motor dysfunction. Cluster 9 contains genes that are increased solely in the only in the 7d group, indicating that these genes may be important later in the course of disease, and perhaps have a role in *maintaining* disease.

**Fig 2:**
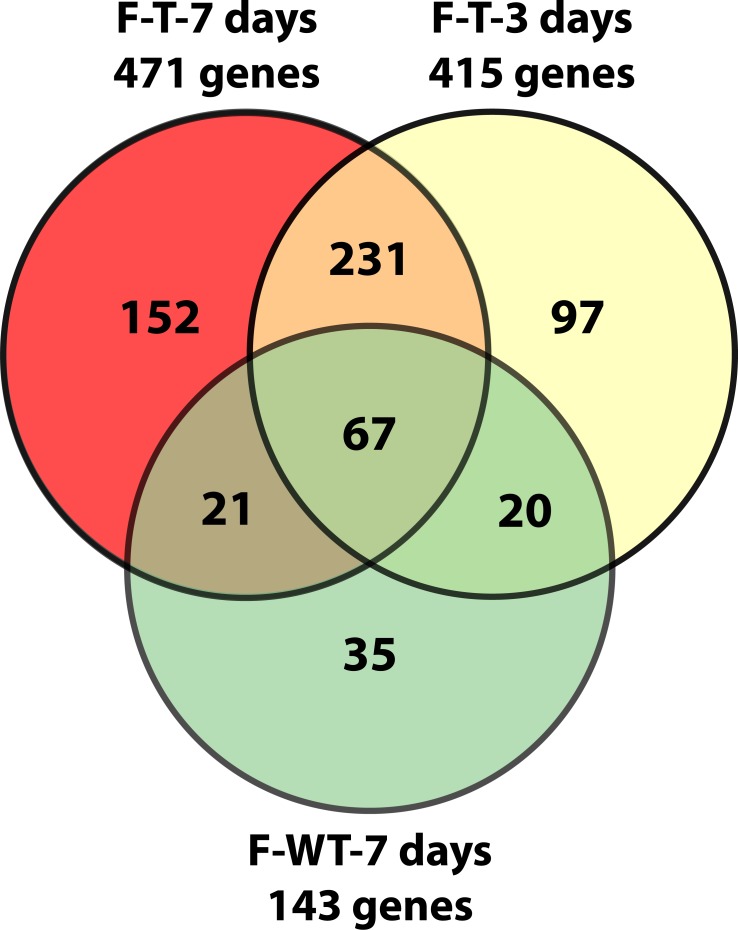
Venn diagram of microarray results in female HSA-AR Tg mice after 3 and 7 days testosterone treatment and WT mice after 7 days testosterone treatment. Diagram representing the number of genes whose expression differed from controls. Gene numbers were obtained by examining gene lists generated by a 2-fold, p < 0.05 criterion.

Fig. 2 shows the relationship of differentially expressed genes in 3 and 7d Tg females and 7d WT females. A total of 108 genes overlap between WT and Tg females, indicating that perhaps expression changes in these genes are a response to T and do not predict deficits in motor function. Nevertheless, there are a total of 231 genes differentially expressed in both 3 and 7d T-treated Tg females, that are not differentially expressed in 7d WT females.

We next performed a functional classification on the differentially expressed genes from Fig. 1 for T-treated Tg females (i.e., clusters 5, 6, 9, 10, and 12) using Functional Annotation Tool (DAVID Bioinformatics Resources) according to biological processes and molecular functions. The functional distribution of the selected genes is listed in Table 1. The analysis indicated that the differentially expressed genes were implicated in a wide variety of gene ontology (GO) functions, including: muscle contraction and development, ion and nucleotide binding, mitochondrion, myofibril, actin skeletal, glucose metabolism, ATPase, neuron development, and vasculature development.

**Table 1:** Functional clustering of differentially expressed genes in female Tg (as compared to WT) with testosterone treatment.

Function	Go Terms	Number of down-regulated genes	Number of up-regulated genes
muscle contraction	regulation of muscle contraction	4	
	sarcoplasmic reticulum	7	
	muscle contraction	13	
mitochondrion	mitochondrion	31	12
	organelle envelope	15	6
	mitochondrial membrane	12	6
glucose metabolism	glucose metabolic process	9	
	glycogen metabolic process	6	
Actin	actin cytoskeleton	10	4
	actin binding	6	6
Myofibril	myofibril	9	6
	sarcomere	8	6
muscle development	muscle organ development	7	6
	striated muscle cell development	4	5
	muscle cell differentiation	4	4
ion binding	ion binding	49	25
	calcium ion binding	20	
	zinc ion binding		17
ion transport	ion transport	13	
	voltage-gated channel activity	6	
	potassium channel activity	3	
ATPase	ATPase activity	4	
	ATP metabolic process	5	
nucleotide binding	nucleotide binding	30	12
	ATP binding	20	12
phosphate metabolism	phosphate metabolic process	10	
	phosphorylation	7	
skeletal development	skeletal system development	5	
	bone development	3	
neuron	neuron/nervous system development	3	4
	neuron differentiation	3	3
	neurogenesis		3
vasculature development	vasculature development		5
	blood vessel development		5
cell adhesion	cell adhesion		7
	biological adhesion		7
proteolysis	proteolysis		10
	peptidase activity		6
oxidation reduction	oxidation reduction		6
	oxidoreductase		4
cell motion	cell motion		4
	cell motility		3
protein transport	protein transport		3
	protein localization		3
apoptosis	apoptosis		4
	programmed cell death		4

**Fig 3:**
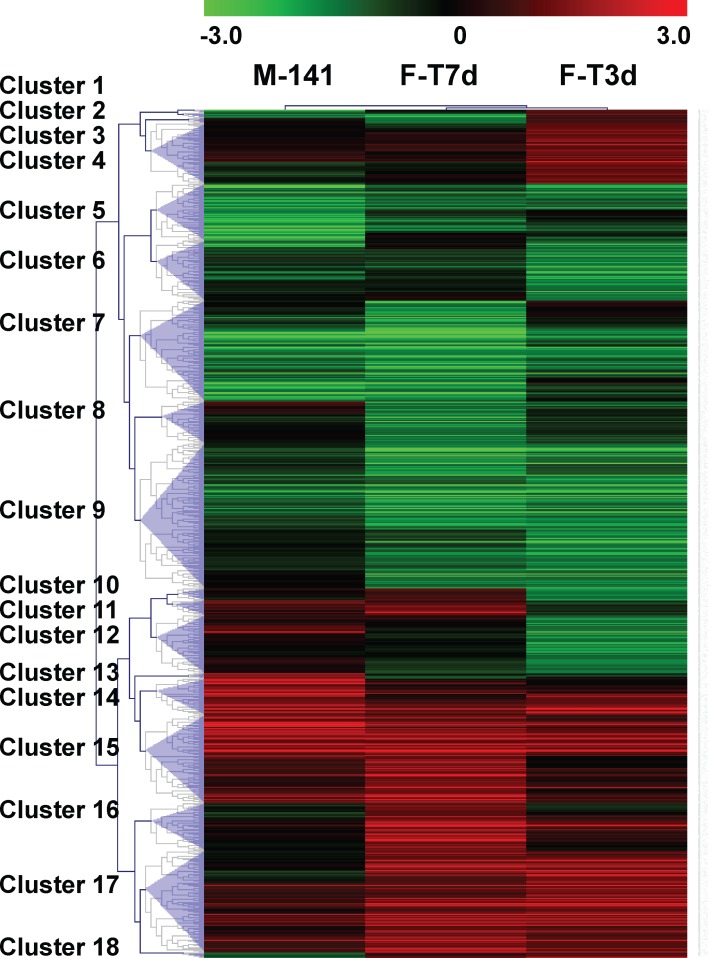
Hierarchical cluster analysis of gene expression in male HSA-AR Tg (M-141) and female HSA-AR Tg after 3 days (F-T3d) and 7 days (F-T7d) testosterone treatment. Cluster output represents colorimetrically indicated log2 ratio change. Male Tg mice and female Tg mice after 3 and 7 days testosterone treatment are represented by columns and rows represent a single gene. Clusters of genes differentially expressed in a similar pattern are labeled.

We then compared gene expression alterations in acutely T-treated Tg females with those we have previously reported for chronically diseased Tg males, which exhibit many similarities in gene expression in skeletal muscle with polyQ mouse models of KD/SBMA [11]. This comparison allows us to identify which of the previously identified candidate genes are androgen dependent. A hierarchical cluster analysis of gene expression in Tg males and 3 and 7d T treated Tg females generated 18 distinguishable clusters (using a criteria of at least 2-fold change and p<0.05; Fig. 3). Each of these clusters is represented with the mean pattern of expression of the genes in each group; down and upregulated genes were found in clusters 5, 6, 7, 9 and 14, 15, 17, respectively. Note that many clusters are differentially expressed in a similar pattern between chronically diseased Tg males and acutely diseased Tg females (clusters 5, 7, 9, 14, 15, 17), suggesting that those disease processes occurring early on may persist, and that these changes may be important in maintaining motor dysfunction. There are also some clusters that show differential gene expression in 3d females (clusters 4, 6, and 12) but not in the more severely affected females (7d treated) or chronically diseased males. Additionally, cluster 2 shows opposite responses between the 3d females and the more affected 7d females and males. The genes that are altered early on in the 3d group may be important for initiating motor dysfunction. Complete gene lists and fold changes are presented in S4 Table.

**Fig 4:**
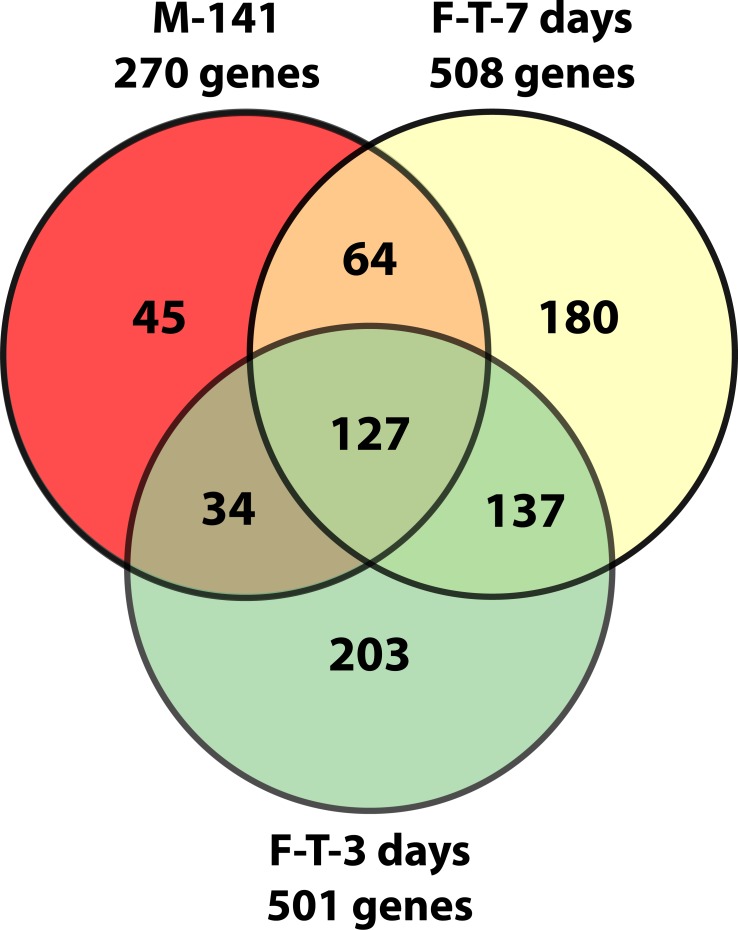
Venn diagram of microarray results in male HSA-AR Tg (M-141) and female HSA-AR Tg after 3 days (F-T-3days) and 7 days (F-T-7days) testosterone treatment. Diagram representing the number of genes whose expression differed from controls. Gene numbers were obtained by examining gene lists generated by a 2-fold, p<0.05 criterion.

Furthermore, examining the overlap between genes differentially expressed after 3 and 7d T treatment in females and Tg males helps us to distinguish between genes which initiate pathology from those that are more likely involved with more chronic wasting or compensatory changes. A Venn diagram shows that at least 127 genes are differentially expressed in all three groups: T-treated Tg females (3 and 7d) and Tg males (Fig. 4).

Another advantage to the current study is that we are able to identify early, transient changes in gene expression following T exposure (i.e., genes that are differentially expressed in 3d treated females but not in 7d treated Tg females and Tg males). There are a total of 203 genes that are differentially expressed specifically in Tg females treated for only 3d, but not differentially expressed in the animals exposed to disease and motor dysfunction for longer. Thus, it is possible that these temporary changes are not critical for maintaining disease progression.

Perhaps the most interesting and valuable comparisons are those between disease-induced Tg female and unmanipulated male KD/SBMA mice (described above). Genes that overlap between the females in the current study and the three male models from Mo et al. [11] lend insight into gene expression changes that are androgen dependent and consistently associated with motor dysfunction. We chose the 7d treated female group rather than 3d as their motor ability is comparable to diseased male KD/SBMA mice. A total of 20 genes were found to overlap across the four groups (Table 2). Note that some of these genes were also differentially expressed in other models like Huntington’s disease (HD; [17]), AR knock out (ARKO; [18]), and Atrophy [19,20] (Table 2). The significance and possible relationship of these candidate genes to the disease process is described further below.

**Table 2:** The list and fold change of differentially expressed genes found in three KD/SBMA mouse models (as compared to WT controls) and female HSA-AR Tg mice with 7 days testosterone treatment (as compared to WT with 7 days testosterone treatment).

ID	Symbol	UniGene Name	Female 141–7d	Male 141	Tg 97Q	KI 113Q	Also in other models
AK009352	Nmrk2	Nicotinamide riboside kinase 2	-9.71	-4.63	-81.97	-13.01	ARKO
NM_008832	Phka1	Phosphorylase kinase alpha 1	-2.85	-5.46	-5.54	-3.95	Atrophy
NM_009505	Vegfa	Vascular endothelial growth factor A	-2.73	-1.86	-5.11	-2.22	
NM_009601	AChRb	Cholinergic receptor (acetylcholine receptor)	2.25	3.20	4.89	2.03	HD
NM_010135	Enah	Enabled homolog	4.76	3.63	3.97	2.01	
NM_010271	Gpd1	Glycerol-3-phosphate dehydrogenase 1	-3.58	-2.49	-7.15	-2.72	HD
NM_010585	Itpr1	Inositol 1,4,5-triphosphate receptor 1	-2.46	-8.72	-3.43	-5.94	HD
NM_013456	Actn3	Actinin alpha 3	-2.38	-4.59	-5.10	-1.93	HD
NM_013602	Mt1	Metallothionein 1	2.22	3.78	2.69	2.45	Atrophy
NM_017379	Tuba8	Tubulin, alpha 8	-2.01	-3.36	-2.71	-2.21	HD
NM_023049	Asb2	Ankyrin repeat and SOCS box-containing protein 2	-5.03	-3.45	-2.28	-2.92	
NM_026633		RIKEN cDNA 9530058B02 gene	-4.44	-2.73	-3.11	-2.54	
NM_030143	Ddit4l	DNA-damage-inducible transcript 4-like	-3.14	-6.45	-6.23	-5.51	
NM_145533	Smox	Spermine oxidase	-3.20	-7.27	-33.20	-15.25	ARKO
NM_153744	Prkag3	Protein kinase, AMP-activated, gamma 3	-3.01	-2.52	-2.51	-2.06	
NM_175031	Stk36	Serine/threonine kinase 36	-2.16	-2.19	-3.41	-3.42	
NM_181390	Mustn1	Musculoskeletal, embryonic nuclear protein 1	5.17	2.92	11.01	5.57	
NM_207530	Osbpl1a	Oxysterol binding protein-like 1A	2.50	3.57	2.51	2.14	
NM_029104	Mss51	Mitochondrial translational activator	-7.36	-9.47	-8.79	-32.22	ARKO
XM_358335	Cacna1s	Calcium channel, voltage-dependent, alpha 1S	-3.43	-2.97	-2.76	-2.08	

Some of these genes were also differentially expressed in other models like Huntington’s disease (HD; [17]), AR knock out (ARKO; [18]), and Atrophy [19,20].

**Fig 5:**
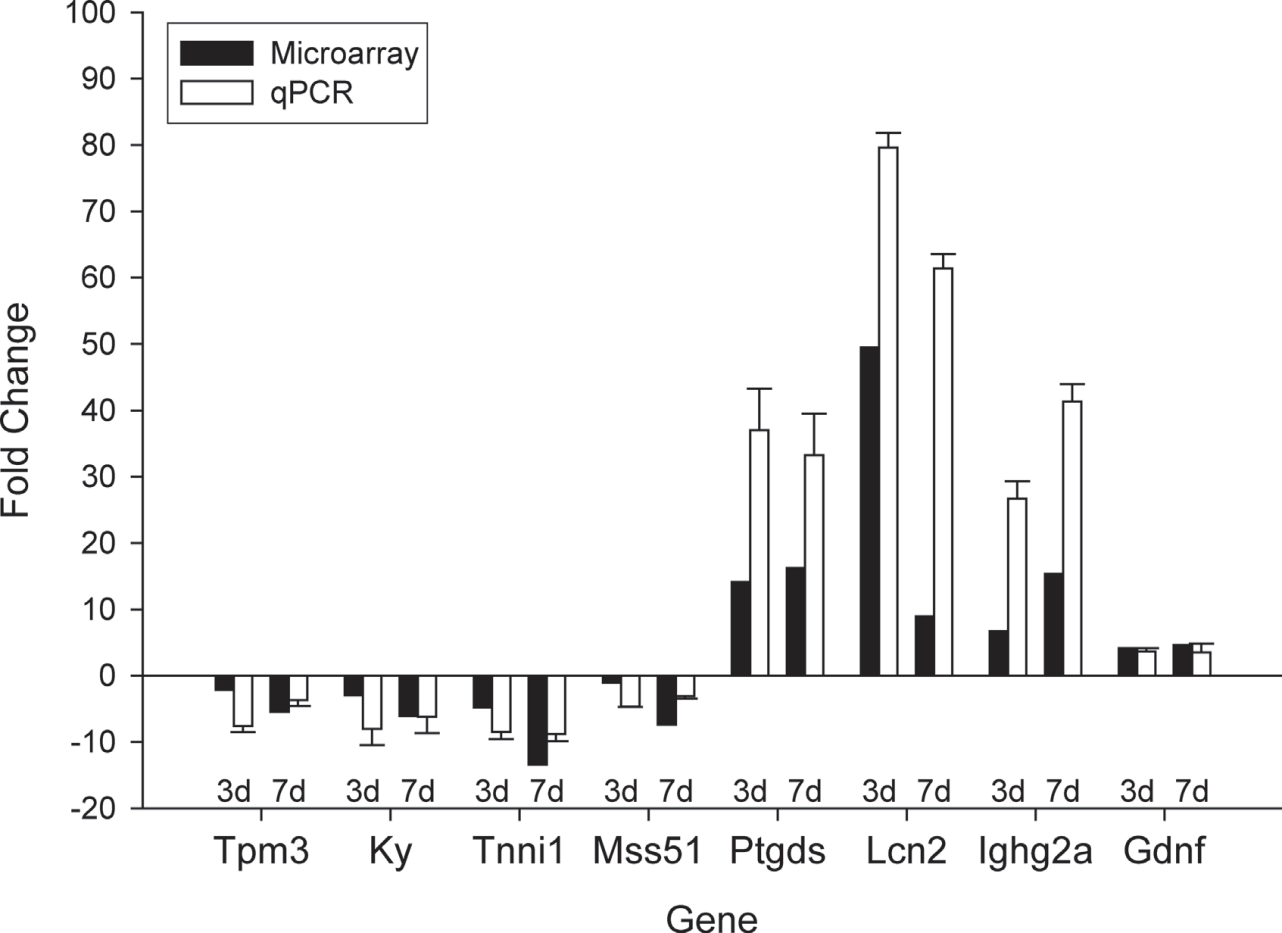
Validation of the results of microarray experiments by qRT-PCR analysis. Overall, we find consistency between qPCR and microarray analysis. Genes of varying fold changes were chosen to demonstrate the validity of microarray data. Bars show Tg females treated with testosterone for 3 days as compared to WT treated for 3 days, and Tg females treated for 7 days as compared to female WT treated for 7 days. Error bars represent standard error of the mean.

Finally, to validate our microarray analysis, we performed quantitative real-time PCR (qRT-PCR) on several important genes with higher, middle, and lower levels of mRNA as detected by the microarray (Fig. 5). Genes included: MSS51 mitochondrial translational activator (*Mss51*), Tropomyosin 3 (*Tpm3*), Kyphoscoliosis peptidase (*Ky*), Troponin I (*Tnni1*), Prostaglandin D2 synthase (*Ptgds*), Lipocalin 2 (*Lcn2*), immunoglobulin heavy constant gamma 2A (*Ighg2a*) and Glial cell line derived neurotrophic factor (*Gdnf*). All samples were compared to WT controls to evaluate fold changes. Although the magnitudes of changes in levels of mRNA were different, qRT-PCR analyses were consistent with microarray results and similar patterns of regulation in female Tg mice with T were observed.

## Discussion

In this study, we examined the transcriptional changes in muscles of females that overexpress *AR* specifically in myocytes. Following 7d of T treatment, motor dysfunction is profound in these mice, comparable to their chronically diseased male siblings [13]. We found that many of the genes dysregulated in muscle of KD/SBMA male mice [11] are also dysregulated in females, indicating that these specified genes are both T-dependent, thus relevant for KD/SBMA, *and* important in early disease progression rather than a byproduct of prolonged disease. A total of 20 commonly differentially expressed genes were identified in 7d T-treated HSA-AR females and three previously characterized male mouse models (HSA-AR, 97Q, and 113Q; Table 2).

Notably, many of the candidate genes are involved in muscle regeneration and/or differentiation. Dysregulation of such genes may explain the inability of these mice to maintain muscle fiber integrity and thus endure motor tests. For example, expression of the *Nmrk2* gene (previously known as *Itgb1bp3*) is decreased. This codes for the protein MIBP and is important for communication with laminin in the extracellular matrix. It is expressed at high levels prior to myoblast fusion and decreases following differentiation [21]. *Asb2* (codes for a subunit of E3 ubiquitin ligase complex) is also downregulated. This gene is induced during myogenic differentiation and a knockdown of it leads to delayed myotube formation [22]. *Stk36* (coding for homolog of the Drosophila Fused gene) is reduced in female and male KD/SBMA muscle. This protein is important in vertebrates as demonstrated by its effects on muscle differentiation in zebrafish, acting via the hedgehog pathway [23]. However, knocking out this gene in mice did not cause apparent detrimental effects [24]. *Smox* is downregulated in all of the male models and in females treated with T for 3 or 7d. The protein coded by this gene, spermine oxidase, is important for maintaining polyamine homeostasis. It is associated with muscle differentiation and increases during late differentiation stages [25]. Furthermore, some diseases are associated with dysregulation of spermine oxidase, and inhibition of polyamine catabolism can be fatal (reviewed in [26]). Additionally, the *Gpd1* gene (coding for glycerol-3-phosphate dehydrogenase 1) is important in lipid biosynthesis and also linked to muscle cell differentiation, being expressed more in adult muscle [27]. We detected decreased expression of *Gpd1* in adult KD/SBMA muscle, indicating a shift towards more myoblasts suggestive of a regenerative response. Alternatively, it may be a side effect of weight loss, as a decrease has been reported in humans following gastric bypass surgery [28].

The *Enah* gene codes for the Mena protein in mammals and is important for cytoskeletal actin dynamics [29]. A recent study demonstrated that overexpression of Mena in cardiac myocytes caused hypertrophy and negatively impacted heart function following injury, leading to contractile dysfunction [30]. We show that expression of *Enah* is increased in skeletal muscle of KD/SBMA mice, which may lead to perturbed myocyte homeostasis and/or regeneration ability. Another gene important for maintaining myocyte homeostasis is *Prkag3*, as it codes for the gamma-3 subunit of the AMP-activated protein kinase (AMPK). Activation of AMPK results in glucose uptake, which is important during muscle contraction. Also, differentiation of myoblasts to myocytes results in increased gamma-3 mRNA. *Prkag3* transcripts were reduced in KD/SBMA muscle, which may lead to altered contraction ability. *Mustn1* transcripts (which code for Mustang) are increased in KD/SBMA muscles of female and male models. Upregulation of this gene occurs during adult regeneration, hypertrophy, and exercise [31,32]. Increased Mustang in KD/SBMA mice may contribute to their ability to recover following T removal [8,9,12,13].

Denervation-like responses are also present in muscles of these KD/SBMA mice. For example, an AchR subunit (alpha) was upregulated in the HSA-AR model [9,13]. The current study revealed that the beta polypeptide 1 subunit (*AchRb*) is also increased, which is also representative of what occurs in the denervated diaphragm and in *mdx* mice [33].

We observed *Vegfa* downregulation in T-treated HSA-AR females in the current study, which is consistent with what was previously reported in male and female KD/SBMA mice [9,11,13,34]. Interestingly, local application of this growth factor at the muscle ameliorates axonal transport deficits in HSA-AR and 113Q male mice [35] and improves disease progression in an amyotrophic lateral sclerosis rat model [36]. The *Osbpl1a* gene, coding for oxysterol binding protein-like 1A (ORP1l), is important for positioning late endosomes [37] and is upregulated in KD/SBMA muscle. Overexpression of ORP1l results in reduced endosome motility [38]. Furthermore, ORP1l interacts with Rab7 [39], a GTPase that is important for vesicle trafficking. Rab7 is important for trafficking neurotrophic factors such as BDNF and its receptor [40] and thus impairments in ORP1l regulation may ultimately deprive the muscle and/or motoneuron of receiving growth factor signals. Additionally, the *Tuba8* gene codes for an alpha tubulin, which makes up cytoskeletal microtubules. Its downregulation in KD/SBMA muscle may contribute to deficits in growth factor transport.

Proteins involved in calcium handing are downregulated in muscle of KD/SBMA mouse models (*Itpr1*, inositol 1,4,5-triphosphate receptor 1 and *Cacna1s*, voltage-dependent calcium channel alpha 1s). Oki et al. [41] examined contractile properties of T-treated HSA-AR females and suggest that deficits in twitch and tetanus kinetics may be due to improper calcium handling. Additionally, we note deficits in transcript levels of *Actn3* (Actinin alpha 3) in KD/SBMA muscle. Actn3 knockout results in reduced glycogen phosphorylase activity in mice and slower calcium handling kinetics in cultured primary mouse myotubes [42]. Decreased expresstion of these three genes may contribute to some of the contractile deficits examined in Oki et al. [41].

In the current study we present data for which androgen-dependent transcriptional alterations occur during KD/SBMA disease onset. It would be interesting in the future to identify transcriptional alterations that occur during recovery (either castration in males or T-removal in females). This will be particularly useful for identifying those genes which mediate recovery. Furthermore, those genes that do not change during early recovery can be ruled out as they are not involved in motor improvements. Likewise, those genes that are differentially expressed prior to any T treatment in Tg females with intact motor function might also be excluded as important in disease progression, as they may be differentially expressed solely due to transgene presence.

## Supporting Information

S1 Table List of primers used for quantitative RT-PCR validation of microarray results. (XLS)

S2 Table Genes altered in transgenic females following testosterone treatment. Genes altered in transgenic muscle following 3D or 7D testosterone treatment. For inclusion, each gene had to appear in the gene list generated by a 2 fold change, p≤0.05 criteria. Fold change values relative to wildtype controls treated for equivalent time are presented, as are Genbank accession numbers, unigene symbols and names.
(XLS)

S3 Table Genes altered in wildtype females following testosterone treatment. Genes altered in wildtype muscle following 3D or 7D testosterone treatment. For inclusion, each gene had to appear in the gene list generated by a 2 fold change, p≤0.05 criteria. Fold change values relative to untreated wildtype controls are presented, as are Genbank accession numbers, unigene symbols and names.
(XLS)

S4 Table Comparison of genes altered in transgenic males with those altered in transgenic females following testosterone treatment. Genes altered in transgenic males relative to wildtype males [11] and transgenic females treated with testosterone relative to wildtype controls treated for equivalent time relative to wildtype muscle following 3D or 7D testosterone treatment. Fold change values, as are Genbank accession numbers, unigene symbols and names.
(XLS)
